# Doctors' Insights into the Patient Perspective: A Qualitative Study in the Field of Chronic Pain

**DOI:** 10.1155/2014/514230

**Published:** 2014-05-18

**Authors:** Claudia Zanini, Piercarlo Sarzi-Puttini, Fabiola Atzeni, Manuela Di Franco, Sara Rubinelli

**Affiliations:** ^1^Department of Health Sciences and Health Policy, University of Lucerne and Swiss Paraplegic Research, Frohburgstrasse 3, 6002 Lucerne, Switzerland; ^2^Swiss Paraplegic Research, Guido Zäch Institute, Guido Zäch Strasse 4, 6207 Nottwil, Switzerland; ^3^Rheumatology Unit, L. Sacco University Hospital, Via G.B. Grassi 74, 20157 Milan, Italy; ^4^Department of Internal Medicine, Policlinico Umberto I, Sapienza University of Rome, Via del Policlinico 155, 00161 Rome, Italy

## Abstract

*Purpose.* To strengthen the conceptualization of the patient perspective by identifying aspects that, from doctors' point of view, are important to address during a consultation to build a partnership with patients. *Method.* Semistructured interviews were conducted with 17 doctors who are experts in the field of chronic pain in Italy. The recordings of the interviews were transcribed verbatim and interpreted using thematic analysis. *Results.* The participants agreed about the importance of doctors addressing aspects of the patient perspective that can lead to a difference of opinion with patients, namely, patients' views about their health condition (i.e., what they think they have and why and the perceived impact of the health condition on their life) and about treatments (i.e., what they have tried or have heard about and their expectations). *Conclusions.* Identifying patients' standpoints on their health condition and treatments offers an opportunity for critical discussion of differences of opinions and promotes communication exchange and agreement about the appropriate course of action.

## 1. Introduction 

In the last two decades, research in the field of patient-centeredness has promoted the concept of the patient perspective as an important focus during a medical consultation [1, 2]. In the doctor-patient interaction today, many patients prefer a shared decision making model, which includes their perspective [3, 4]. Studies show that integrating the patient perspective has the potential to increase the patient's satisfaction with the consultation [3–5], as well as resulting in better decisions and in improved management of the illness and health outcomes [6, 7].

Despite claims postulating the importance of including the patient's perspective, several constraints on the medical consultation play a role in how doctors respond to the need to include this perspective. Time constraints are a key issue in today's consultations, and doctors, based on the assumption that letting patients speak increases the length of the consultation, often decide to follow their own agenda and interrupt patients after the expression of their first concern [8, 9]. Patients often have more than one concern per visit [10, 11]. By focusing the information exchange on the medical agenda and stressing symptoms and clinical history, doctors may miss significant patient concerns [12–15].

Despite the emphasis of the literature on the value of the patient perspective, the concept of the patient perspective is unclear, as are the aspects of the patient perspective that need to be addressed in the consultation. Indeed, the notion of the patient perspective remains vague and fragmented [1, 2, 12].

Thus far, the patient perspective has been examined in terms of the point of view of patients and in terms of what patients think is important that doctors address during the consultation. Based on the above, the patient perspective has been defined as the self-perceived impact of the health condition on their life [16], as their expectations of the consultation or the doctor [17–19], and as their priorities regarding the outcomes of the treatment [20, 21]. All these aspects have been explored in various settings (e.g., general practice [22], oncology [23], and end of life care [24]).

In this paper, we are interested in examining doctors' perspective of what aspects of the patient perspective matter most. The identification of doctors' insights into this issue is essential, in parallel with patients' insights, for operationalization of the concept of the patient perspective. In light of this, the objective of this paper is to advance understanding of the patient perspective by identifying aspects that, from the point of view of doctors, are important to address to build a partnership with their patients.

## 2. Methods 

This paper presents the results of a qualitative study conducted in Italy, based on semistructured interviews with 17 doctors who are experts in the field of chronic pain. Chronic pain is a particularly relevant area to examine the patient perspective because patients are exposed to health information in different settings and from different sources [25] and because they have to manage their health condition on a daily basis. Participants in the study were recruited through purposive sampling of Italian experts active in the field of fibromyalgia and chronic widespread pain. They were invited to take part in the study during the “Giornate Reumatologiche Sannite,” a major Italian event dedicated to education and exchange among specialists treating rheumatologic patients. The characteristics of the sample are as follows:* gender*: six women (35.3%) and 11 men (64.7%);* age*: ranging from 34 to 73 years (mean of 54);* years of practice*: ranging from nine to 40 (mean of 27);* specialty*: rheumatology (*n* = 12; 70%), neurology (*n* = 2; 11.8%), immunology (*n* = 1; 5.9%), psychiatry (*n* = 1; 5.9%), and nervous and mental disease (*n* = 1; 5.9%);* region of practice*: Northern Italy (*n* = 6; 35.3%), Central Italy (*n* = 3; 17.6%), Southern Italy (*n* = 8; 47%); and* type of employment*: public hospital (*n* = 11; 64.7%), private practice (*n* = 3; 17.6%), and public hospital and private practice (*n* = 3; 17.6%).

The interviews were informed by earlier investigations in the field of doctor-patient argumentation [26], as well as by input from doctors taking part in the study. Being aware of the many problems of communication in the field of chronic pain, we started the semistructured interviews by generally asking doctors about the communication with their patients. Then, we asked more specifically why some patients are considered to be more difficult than others and what the characteristics of a “difficult patient” are. Through examples from their practice, doctors were able to sketch a profile of a difficult patient, and to highlight main aspects to take into consideration to ensure partnership and continuity of care.

One researcher (Claudia Zanini) conducted all the interviews. Due to the extensive experience that the participants have amassed in medical consultation during their long practice, no further interviews were required as data saturation was achieved. The interviews were audio recorded and transcribed verbatim. A number of key themes were identified through a thematic and comparative analysis approach [27]. Our conceptualization of the patient perspective for the medical consultation is primarily based on the coding of those examples drawn from the doctors' experiences, in which the participants identified the characteristics of difficult patients and some challenges and strategies to build a partnership with them. The thematic analysis was used to go beyond the individual experience of every participant and to build a rich description of the data. Themes identified were then grouped together, moving from specific themes to more abstract ones. The data were then checked for consistency. To eliminate bias, two of the authors (Claudia Zanini and Sara Rubinelli) met on a regular basis to verify the data and ultimately reach consensus.

## 3. Results 

Overall, the present study revealed that, especially in the field of chronic pain where doctors do not have a “magic wand” to solve the problem, patients develop strong views regarding their health. In light of this situation, the participants emphasized the importance of addressing the views that patients formed before the medical consultation. More specifically, the doctors highlighted the importance of considering aspects of the patient perspective that can lead to a difference of opinion with patients. These include the patients' perspectives of their health condition and the treatments they have tried or they know about. Furthermore, asking patients about their views and past experiences was considered as a way of building a partnership with the patient by “showing an interest in the person rather than just his or her symptoms.” (female, 34 y/o, rheumatology).

### 3.1. Patients' Perspectives of Their Health Condition

One of the main issues that can lead to a difference of opinion between doctors and patients is the patients' beliefs about their health condition and its causes (i.e., what they think they have and why and the self-perceived impact of the health condition on their life). Thus, for instance, if a patient comes to the consultation thinking that he/she suffers from a specific condition.

“It is very important to listen to what the patient thinks he/she has and, if the patient is wrong, to explain why he/she is wrong. Otherwise, the patient will hold on to that idea always.” (female, 56 y/o, psychiatry).

Another source of conflict may occur in diagnosing the source of the patient's condition, with patients fully convinced that an additional examination (often identified through an Internet search) will determine conclusively the cause of their problem and allow them to choose an effective treatment. Agreeing to an additional medical examination can be beneficial to the doctor-patient interaction because it lowers patient anxiety and shows the doctor's commitment to elucidating the cause.

“Sometimes patients refuse to believe that their condition cannot be treated with antibiotics. They search the Internet and insistently ask for a series of medical investigations that you, as a doctor, would not perform. In such cases, a medical investigation can work as a therapy because it helps calm the patients and at the same time shows that you listened to them.” (male, 63 y/o, neurology).

Similarly, it may be difficult for patients to accept that the cause and the development of their health problem are beyond their control, such as in case of a degenerative problem due to ageing or their genes.

“There are a lot of myths and misconceptions about back pain. The most common is that back pain is the result of physical effort. When you try to make clear that the pain is not due to the fact of being a construction worker, but to genes, patients are often unwilling to accept this explanation because it means they have to accept that there is no definitive solution for their condition.” (male, 48 y/o, rheumatology).

However, agreement on the definition of the problem and its causes is essential because it results in higher motivation to engage in a treatment plan.

“If patients understand what they suffer from and why, it is more likely that they will become active and collaborate in treating their problem.” (female, 55 y/o, rheumatology).

Moreover, it is important not to underestimate the patient's self-perceived effect of the condition and its burden on their life, as this can lead to dissatisfaction with the consultation.

“Someone has back pain and says that it is okay. Someone else has back pain and says it is a catastrophe. It is important to understand the burden of the problem in the patient's life. If you do not address what matters to patients, there is a risk that they will search for a second opinion.” (male, 57 y/o, rheumatology).

Finally, supporting patients in the resolution of problems associated with their condition (e.g., sleeping disorders or dizziness) that they consider place additional burdens on their life contributes to the building of the doctor-patient partnership because it shows an interest in the patients' concerns.

“Maybe you think that this is no big deal from a medical point of view, but if the patient repeats it again and again and even says that it matters to him/her, then it is worth suggesting a therapy for this problem as well. This means investing in a partnership with your patient.” (male, 56 y/o, rheumatology).

### 3.2. Patients' Perspectives of Treatments

The second crucial element that emerged from the interviews concerns patients' perspectives of treatments. Overall, the participants acknowledged that patients should be guided in autonomously deciding to accept a treatment. This guidance requires that doctors explain and justify a particular course of action.

“We have to ‘sell' our solution, give reasons, play our cards right. Patients have to embrace our suggestion because they are convinced that it is the right one and not because we want them to choose a particular option.” (female, 43 y/o, rheumatology).

Although doctors have documentation about their patients' clinical histories, to facilitate autonomous decision making it is important to ask patients about treatments that, in their view, worked or did not work, as well as whether they have heard something positive or negative about treatments that they have not yet tried.

“When treating chronic patients who have tried many therapies and have not found a solution, you have to listen to them and ask what they have already tried and what they have heard or read about a treatment. If you propose something that is inconsistent with their experience or knowledge, there is a risk that they will not listen to you.” (male, 73 y/o, rheumatology).

Moreover, by identifying patients' expectations with regard to treatments, doctors can tailor their proposals to the individual patient or explain to patients why their wishes are inappropriate.

“Some patients simply want to be treated. Others are more specific and want pills or injections or a more psychosomatic approach. It is important for doctors to understand what they think about treatments and what expectations they have. Sometimes we can go along with their wishes, but sometimes we need to explain to patients why their expectations about treatment efficacy are not appropriate.” (male, 57 y/o, neurology).

Paying attention to patients' ideas and expectations about treatment is crucial to avoid nourishing false hopes, as well as to detect gaps between hopes and “what doctors can realistically offer.” (male, 53 y/o, immunology). When there is no “miraculous medication,” doctors should clarify that the proposed treatment is tentative, that other possibilities exist if the suggested remedy does not work, and that giving up the treatment is also an option.

A last point mentioned by the doctors that could be a source of disagreement between the doctor and the patient about the treatment proposal is patients' beliefs about side effects. Doctors need to check the correctness of these beliefs to make sure that patients are not refusing a treatment proposal or giving up a treatment for the wrong reason. A typical example among chronic back pain patients is the link between a needle introduced in the back and additional or new pain.

“Patients always link a medical act, such as a needle in the back, with undesired consequences. Although a needle in the back is invasive, it does not touch the spinal cord, and it does not have the side effects that they sometimes imagine.” (male 54 y/o, rheumatology).

To sum up, together with eliciting patients' past experiences and expectations about treatments, doctors should provide clarity about treatment options and side effects as part of a good doctor-patient interaction.

## 4. Discussion and Conclusion

This study highlights aspects of the patient perspective that, from the doctors' point of view, are important to address in building partnerships with their patients. Two main components of the patient perspective emerged: patients' views about their health conditions (i.e., what the condition is, what its causes are, and how serious it is according to the patient) and patients' views about treatments (i.e., what treatments worked/did not work, if there is any treatment they have heard about, and their expectations with regard to recovery or management).

This study contributes to the advancement of current literature on the patient perspective in medical consultations for five main reasons.

First, the findings of this study enrich the list of topics that are advisable for doctors to discuss with their patients during a medical consultation. Addressing the patient perspective is perceived as an essential step to enhance patient participation, to build agreement through a critical exchange between doctors and patients, and finally to contribute to patients' satisfaction [28, 29].

Second, in recent years, various streams of research have highlighted different components of the patient perspective in a wide range of areas, including the nature and impact of specific conditions [16, 30], the evaluation of specific health-related interventions [31, 32], preferred modalities of communication [19, 33] and consultations [18, 21], and the development of outcome assessment [34]. In relation to this research, the present study points to the importance of the concept of agreement between the doctor and the patient and of the communication process that leads to such agreement. It addresses ways to reach agreement by reflecting on what can give rise to a difference of opinion between doctors and patients. Differences of opinion left unaddressed can become a matter of (hidden) disagreement with patients and even degenerate into conflict [35–37].

Third, our findings are consistent with different conceptualizations of the patient perspective from the patients' point of view [16–21]. There seems to be a partial convergence of interests between patients and doctors with regard to the aspects that they think are important to discuss, namely, the burden of the health condition on patients' life, patients' expectations, and patients' preferences for treatment. This convergence strengthens the relevance of our study and points to the development of strategies and instruments to integrate the patient perspective into medical consultation.

Fourth, the active involvement of patients through the aspects identified within our study naturally links to the growing body of research that Gardiner [38] calls the transition from “informed patient care” to “patient informed care.” In the age of information, patients form their views outside the medical consultation, and these views can facilitate or hinder any collaboration with doctors [39, 40]. For patient informed care to take place, doctors also need to identify and address aspects of the patient perspective that may be different or inconsistent with their own.

Fifth, this study supports recent research [26] that suggests looking at the medical consultation as an argumentative exchange where doctors and patients may have different perspectives. It ultimately points to the value for doctors and patients to engage in argumentation, defined as the communication process of exchanging points of view with the aim of resolving a difference of opinion [41].

### 4.1. Implications for Research

The present study contributes to the conceptualization of a patient perspective that is instrumental to the medical consultation but leaves unanswered the question of how to integrate it. Research has shown that instruments such as prompt sheets can be of help during the medical consultation [42, 43]. However, empirical research is needed to identify best practices in specific contexts.

Existing studies also highlight topics that doctors consider to be threatening to the relationship or disruptive to schedule and medical agenda. For instance, doctors tend to ignore cues and concerns to negative emotions and focus the discussion on medical issues [44]. However, emotions (e.g., fear and anger) or worry about the health condition (e.g., its severity) or treatments (e.g., side effects) are often embedded in the patients' views and—as shown in our findings—addressing them can be beneficial not only for the relationship but also for clinical purposes (e.g., for tailoring plans of care) [44]. Ultimately, research on the challenges of the integration of the patient perspective, as well as on the strategies to deal with them, could lay out the basis for a more reflective practice and inspire the development of specific training courses for doctors.

### 4.2. Limitations

We present here a qualitative study that, as such, does not have generalizability as a goal. Our findings may not be valid for the field of acute care, where patients have less time to develop a strong perspective, where there is often a specific treatment that solves the problem in a short time and there are fewer opportunities for critical discussion and exchange of views. However, the present study offers a rich insight into the unique nature of doctor-patient interaction in the field of chronic pain, a field where patients develop a strong knowledge of their health condition and know-how for its management.
